# Opening the debate on deep brain stimulation for Alzheimer disease – a critical evaluation of rationale, shortcomings, and ethical justification

**DOI:** 10.1186/s12910-018-0275-4

**Published:** 2018-06-11

**Authors:** Merlin Bittlinger, Sabine Müller

**Affiliations:** Charité – Universitätsmedizin Berlin, corporate member of Freie Universität Berlin, Humboldt-Universität zu Berlin, and Berlin Institute of Health, Department for Psychiatry and Psychotherapy, CCM, Division of Mind and Brain Research, Charitéplatz 1, 10117 Berlin, Germany

**Keywords:** Deep brain stimulation, Alzheimer disease, Fornix, Nucleus basalis of Meynert, Ventral capsule/ventral striatum, Risk-benefit assessment, Safety, Evidence-based hypothesis-driven research

## Abstract

**Background:**

Deep brain stimulation (DBS) as investigational intervention for symptomatic relief from Alzheimer disease (AD) has generated big expectations. Our aim is to discuss the ethical justification of this research agenda by examining the underlying research rationale as well as potential methodological pitfalls. The shortcomings we address are of high ethical importance because only scientifically valid research has the potential to be ethical.

**Method:**

We performed a systematic search on MEDLINE and EMBASE. We included 166 publications about DBS for AD into the analysis of research rationale, risks and ethical aspects. Fifty-eight patients were reported in peer-reviewed journals with very mixed results. A grey literature search revealed hints for 75 yet to be published, potentially enrolled patients.

**Results:**

The results of our systematic review indicate methodological shortcomings in the literature that are both scientific and ethical in nature. According to our analysis, research with human subjects was performed before decisive preclinical research was published examining the specific research question at stake. We also raise the concern that conclusions on the potential safety and efficacy have been reported in the literature that seem premature given the design of the feasibility studies from which they were drawn. In addition, some publications report that DBS for AD was performed without written informed consent from some patients, but from surrogates only. Furthermore, registered ongoing trials plan to enroll severely demented patients. We provide reasons that this would violate Art. 28 of the Declaration of Helsinki, because DBS for AD involves more than minimal risks and burdens, and because its efficacy and safety are not yet empirically established to be likely.

**Conclusion:**

Based on our empirical analysis, we argue that clinical research on interventions of risk class III (Food and Drug Administration and European Medicines Agency) should not be exploratory but grounded on sound, preclinically tested, and disease-specific a posteriori hypotheses. This also applies to DBS for dementia as long as therapeutic benefits are uncertain, and especially when research subjects with cognitive deficits are involved, who may foreseeably progress to full incapacity to provide informed consent during the required follow-up period.

## Background

Deep Brain Stimulation (DBS) is an invasive neurosurgical procedure. A small burr hole is driven into the skull and thin electrodes are inserted deep into specific brain targets to stimulate the tissue electrically. The stimulation parameters (frequency, voltage and pulse width) can be adapted to either optimize the outcome symptoms or to minimize potential side effects. DBS has been used in more than 100.000 patients. It is approved by the U.S. Food and Drug Administration (FDA) for symptomatic motor improvement in severe Parkinson’s disease (PD) and dementia is one of the main exclusion criteria. DBS received also FDA approval under a Humanitarian Device Exemption for the indication obsessive-compulsive disorder [1]. The question how research on DBS for new indications should be regulated and which ethical requirements need to be fulfilled is a hot topic in current bioethics [2–6].^1^

In the present article, we critically examine the research rationale for DBS in Alzheimer disease (AD) from an ethical point of view with close focus on aspects of scientific validity. We start by summarizing the context of discovery that has led to the research idea to relieve AD symptoms with DBS. Afterwards, we systematically examine the context of justification by scrutinizing the research rationale as well as relevant open question and unaddressed potential risks from an ethical perspective.

### DBS for AD research - context of discovery

In 2008, Hamani and colleagues treated one patient with DBS with the aim to reduce his morbid obesity. The stimulation induced déjà vu-like episodic memory flashbacks. This “unanticipated collateral effect” was accompanied by stimulation-dependent improvements in the California Verbal Learning Test after 12 months [7]. The authors suggested that this effect is “consistent with driving the activity of the hippocampal memory circuit through stimulation of the fornix” [7]. Furthermore, they speculated that “it may be possible to apply electrical stimulation to modulate memory function and, in so doing, gain a better understanding of the neural substrates of memory” [7]. Even though the effect on memory was clinically irrelevant for the research subject, the authors presented this case as hope for “memory enhancement”. However, commentators rightly remarked that “such reminiscences are dysfunctional phenomena, because they occur in an uncontrolled and involuntary manner that is not useful in guiding behavior” [8]. The report rather portrays an unsuccessful attempt to treat morbid obesity but this was only discussed as such in the Supporting Information in the appendix.^2^

In 2004, the International Committee of Medical Journal Editors pointed out that single case reports may give rise to the problem of selective-reporting [9]. The serendipitous finding of Hamani and colleagues may be seen as a good example for this potential risk. Similarly, a single case of DBS of the Nucleus basalis of Meynert (NBM) was reported to improved apraxia in PD dementia [10, 11]. In spite of the differences in clinical physiology between PD dementia and AD, which make a direct translation from one indication to the other challenging, this case was reported to have “formed the basis” [12] to explore DBS of the NBM in AD.^3^ Although DBS research is considered to be prone to selective reporting by some DBS researchers [13], research with human AD patients was apparently pushed forward on the basis of single case reports from other indications. In the very first scientific reports about DBS for AD, there is little mention of additional empirical evidence for any hypothetical benefits of DBS for AD [14–16] and the mentioned findings from PD dementia and morbid obesity are not disease-specific to AD. Reading these reports with close attention to the research rationale, it seemed that speculative interpretation of the “unanticipated collateral effect” in one obese patient [7] was straightforwardly transformed into the optimistic hypothesis “that it might be possible to use DBS of the fornix to drive its activity and to modulate the circuits mediating memory function in patients with […] mild AD” [14]. Further assessment of the context of discovery made it appear to us that this hypothesis was deemed ready to be directly tested in humans, despite that literature reviews reported that available preclinical evidence “cannot be extrapolated to the dementia-like states” [17] and more specific research “to address the effects and mechanisms of DBS in memory deficits” [17] would still be required. Based on this observation, we performed a comprehensive ethical evaluation of the research rationale of DBS for AD to systematically examine its justification.

In recent years, clinical research on DBS in AD patients has been performed mainly by two research groups: First, the group of Lozano and colleagues in Canada, who initiated DBS for AD with their failed attempt to treat obesity. They performed a pilot study with six patients [14] and a pivotal study with 42 patients in a multicenter study including centers from Canada and the USA [18]. Second, the Germany-based group of Kuhn and colleagues who examined eight patients [15, 19]. Whereas the Canadian group stimulates the fornix, the German group stimulates the nucleus basalis of Meynert (NBM). Recently, a third group in the USA started to examine three patients and stimulated a region in the frontal lobe (ventral capsule/ventral striatum) as another potential brain target for AD [20]. Additionally, further trials on fornix DBS are planned or ongoing in China [21] and a Brazilian neurosurgeon already applied fornix DBS in clinical practice.^4^

In spite of the apparent paucity of directly relevant preclinical evidence as reported by literature reviews [22],^5^ the Lozano research group launched the first pilot study with six human subjects in 2008 [14]. The results were considered “not conclusive for clinical outcome” by peers working in the field [17]. Notably, in our survey among 113 dB experts from 12 countries, the prospects of success with regard to DBS for AD had been evaluated with skepticism from the outset [23]. Notwithstanding these skeptical voices, the Lozano research group reported the mixed results of their pilot study as success that could emerge “as viable, potentially beneficial treatment modalities for AD” [24]. Notably, the principal investigator, Andres Lozano, is also founder of the company Functional Neuromodulation Ltd. [25] and co-inventor of a US patent on fornix DBS for AD [26]. The role of such patents for neurotechnological research is currently actively debated [27]. In our opinion, further assessment by ethicists and legal scientists is needed to evaluate the potential effects on scientific progress. In an influential public talk, Andres Lozano declared that his research group decided to “turbo-charge the memory circuits in the brain” and that they “have chosen to treat patients with Alzheimer’s disease” [28]. The announcement to “turbo-charge” memory was made before the scientific publication of conclusive results [18, 28]. Unfortunately, the later clinical trial does not seem to corroborate the evidence for statistically significant and clinically meaningful effects of DBS for patients with AD for primary outcomes [18].

### DBS for AD research – Context of justification

Both FDA and European Medicines Agency (EMA) require that pharmacological trials on risky but yet unproven treatments are based on adequate scientific foundations including preclinical research [29]. From a regulatory perspective, DBS is a medical device belonging into FDA’s and EMA’s class III of implants with highest risks [30–32]. As a medical device, however, it is less strictly regulated than medicinal products like pharmaceutical agents. From an ethical point of view, these legal differences are less relevant. For instance, it may be argued that a bioactive, invasive device intervening systemically in the human brain to alter cellular processes and brain circuits should be subject to just the same ethical standards of clinical research like pharmaceuticals.^6^

In the following sections, we examine the “clinical readiness” of DBS for AD. We systematically assess whether experimental research on brain implants (FDA’s and EMA’s risk class III) like DBS for AD is yet ready for clinical testing from an ethical perspective. Most of the points concern scientific methodology, but this does not question their ethical relevance [33]. Bioethicists should not too readily accept some kind of division of labor assuming that experts in each field know best. For the ethical evaluation of DBS for AD, it is important to critically deal with scientific matters in depth and detail. There is a tight connection between ethics and good scientific methodology: ethical research presupposes scientific validity [34].

The core of scientific validity is a combination of the existing evidence, the hypothesis and the study protocol. To assess clinical readiness, we must therefore ask the following complex question: What exactly is the evidence for the hypothesis that DBS in patient cohort *C* with stimulation parameter settings *S* applied to brain target *T* effects some physiological change, which is with probability *P*_*1*_ positively correlated with clinically relevant outcome *O* and which is with probability *P*_*2*_ not positively correlated with any significant harm that outweighs *O*?

We conducted a systematic analysis of the information published in the scientific literature. The analysis revealed that this question is still open on the basis of available evidence. Thus, any judgement on the relevant probabilities *P*_*1*_ of potential benefits and *P*_*2*_ of potential harms is currently still speculative. We argue that the risk resulting from not sufficiently knowing these probabilities undermines rational and well *informed* decision making as well as adequate risk-benefit assessment and risk mitigation. Therefore, we call for additional basic science, preclinical research and high quality systematic reviews thereof to settle relevant open questions.^7^ In particular, there is still significant uncertainty with regard to how well previous findings from one patient cohort (e.g. single patients with morbid obesity or PD dementia) translate to different patient cohorts presenting diverse clinical symptoms and variance in neuropathology.

To be clear, the question of clinical readiness is not only a matter of scientific facts but involves a normative judgment on what degree of uncertainty about potential harms is acceptable. This can be conceptualized on a continuum ranging from “scientific adventure” involving explorative research on risky interventions to “scientific prudence or caution”. The latter proceeds strictly evidence-based and hypothesis-driven while potential risks are mitigated through pre-clarification of open question with milder scientific means or preclinical research.

Whereas the question of how much evidence is available can be answered by scientific means (e.g. systematic reviews and meta-analyses), the question where to draw the line is irreducible normative. Neuroethical debate is required to discuss the moral reasons that are supposed to justify how the line of demarcation is set.

Currently, the established ethical standards are framed by international conventions like the Declaration of Helsinki of the World Medical Association [35], the ethical guidelines of the Council for International Organizations of Medical Sciences [36], the Convention of Biomedicine of the European Council [37] and various national adaptations. Our ethical evaluation of DBS for AD will draw from these conventions, although one should notice the caveat that other neuroethicists may well defend other positions with reasons.

In the present context, we assume that the line of demarcation is adequately set by the Declaration of Helsinki:

“Medical research involving human subjects may only be conducted if the importance of the objective outweighs the risks and burdens to the research subjects.” (Art. 16, [35]).

Note that the meaning of research objective in Art. 16 above is not any general and speculative goal like finding an effective therapeutic AD treatment in the long run. What is meant by research objective is the particular research question that may be answered by a given study in a scientific valid way [33]. In early first-in-human studies, these research objectives depend crucially on study protocol and are typically questions of feasibility. Here are some examples: Is it technically feasible to insert the electrode into the preselected brain target with sufficient precision given the brain atrophy and cerebrovascular alterations of AD? Is the insertion of the electrodes into deep brain regions like fornix or NBM similarly safe in diverse patients with AD brain pathology as in other diseases (e.g. PD or OCD)? Is an innovative surgical approach needed to reach the intended brain target and is this new approach safe (e.g. the required transventricular electrode trajectory [38] to reach the fornix)? Given that the stimulation is nearby the hypothalamus, which controls neuroendocrine and autonomic nervous function, is the stimulation within the range of no or well-tolerable side-effects biologically active in the antecedently hypothesized way? Is it feasible to perform the very same procedure with sufficient precision, standardization and consistency in a large enough number of patients to draw statistically valid inferences? Is it feasible to successfully recruit, operate and stimulate an adequately powered sample of patients so that the evaluation of the procedure’s risk and efficacy profile is sensitive and reliable?

Although these feasibility questions are of high importance for progressing ethically from the preclinical phase to proper clinical testing, it is also clear that none of the respective answers is directly relevant to the patients who volunteer in such nontherapeutic feasibility studies [29]. With no realistic direct benefit in prospect, the careful risk mitigation becomes even more central*.* Consequently, we must address the question what are the known risks and are there potential unknown risks?

From an epistemological point of view, it is necessary to distinguish between the “negative” *absence* of evidence for any potential harm and the “positive” *evidence* for the absence of harms [39]. Therefore, we hold the following view: If patients with compromised capacity to consent are enrolled in highly complex research with non-minimal risks (Table 1), then researches have highest duties to actively gather relevant empirical information on potential risks. This includes disease-specific preclinical research with the primary goal to systematically rule out hypothetically harmful effects. To avoid a potential misunderstanding, asking such critical questions is very different from being *risk averse*. It is a constructive method to foster *risk awareness*.

**Table 1:** Known and unknown physical risks of DBS for AD

	Risk [*brain target:*], percent range depending on source	Degree of evidence	First author, year, reference
General risks of device implantation	Hemorrhage (1.1–2.5%),	*Indirect evidence* from other indications, not directly translatable to new indications like AD.	Ponce, 2016, [103] Binder, 2003, [104] Fenoy, 2014, [105] Sillay, 2008, [106] Wang, 2017, [107] Voon, 2008, [108] Chhabra, 2010, [109] Morishita, 2013, [110] Saleh, 2015, [111]
	Wound infection (1.7–8%),		
	Hardware failure (1.5–36%),		
	Suicide (0.5%)		
	Encephalomalacia (4.2%)		
	Venous thrombosis (1.3%)		
Special risks of stimulation or device implantation in brain tissue with AD pathophysiology	*Fornix*: Infection (4.8%), lead repositioning (2.4%), chronic subdural hematoma (2.4%), encephalomalacia (2.4%)	*Preliminary evidence* from serial case studies (NBM and VC/VS) or small trials (fornix). *Limited by the lack of sufficient statistical power* to detect adverse effects that are not extremely common; no extrapolation possible in scientific valid ways.	Ponce, 2016, [103] McMullen, 2016, [112] Kuhn, 2015 [15] Scharre, 2016, [20]
	*NBM*: hardware malfunctioning requiring surgical revision (33%)		
	*VC/*VS: no adverse events reported		
Side effects limiting the range of possible stimulation parameters to find beneficial physiological effects	*Fornix*: autonomic and cardiovascular effects including sensations of warmth and increases in heart rate and blood pressure (at high stimulation settings)		Ponce, 2016, [103]
	*NBM:* Transient inner restlessness at higher stimulation intensities of > 5 Volt		Kuhn, 2015 [15]

Definitions**:** An ‘adverse effect’ is an “unfavorable outcome that occurs during or after the use of a drug or other intervention but is not necessarily caused by it” [39]. A ‘side effect’ is any “unintended effect, adverse or beneficial, of a drug that occurs at doses normally used for treatment” [39]

**Fig. 1:**
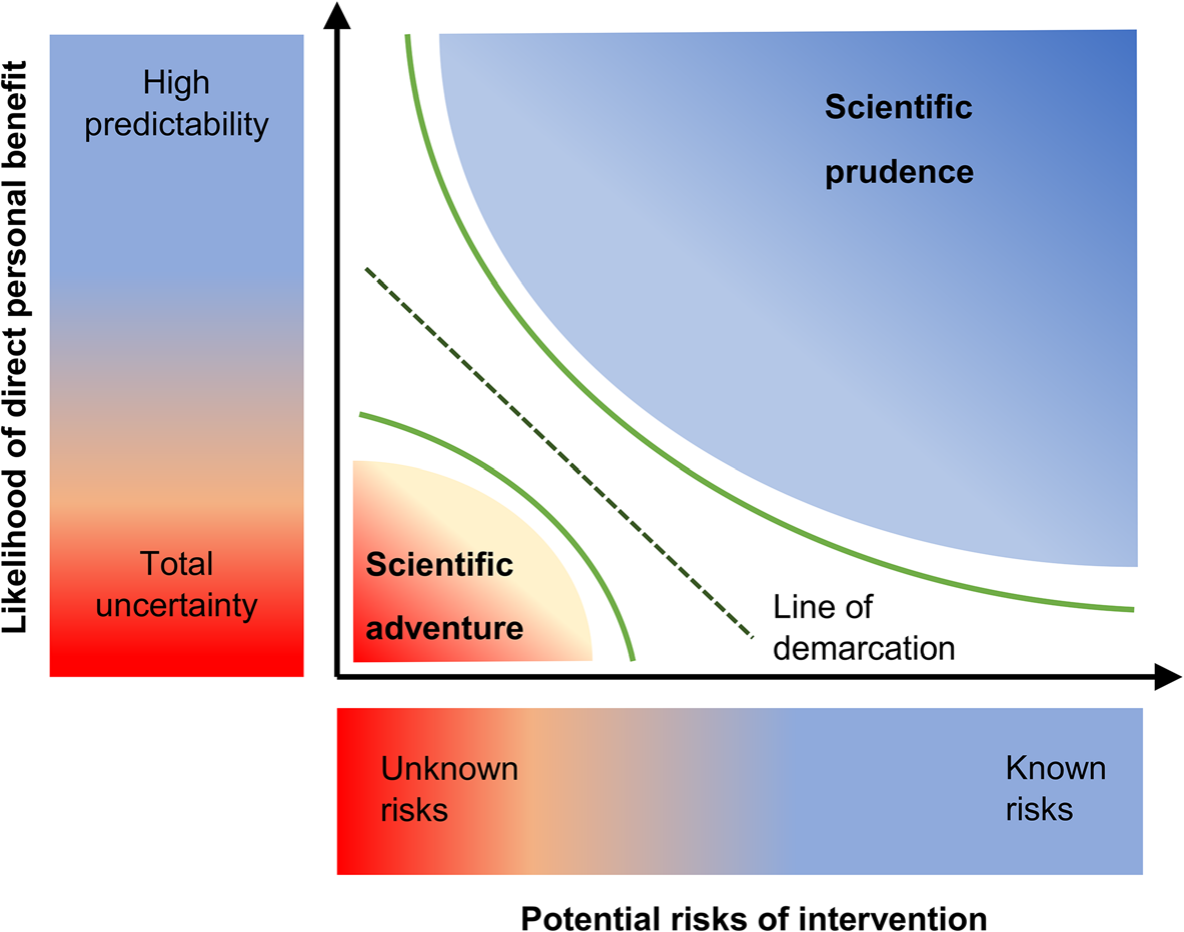
The continuum of any risk-benefit assessment – there is a line of demarcation that separates scientific prudence from scientific adventures

The present article is a systematic examination of research rationale, risk assessment and interpretation of results. We will *retrospectively* examine the question whether recently published trials were sufficiently evidence-based. After that, we evaluate *prospectively* whether the interpretation of results is adequate to inform future evidence-based decision making. With the continuum still in mind (Fig. 1), our aim is to pose *relevant* critical questions that open a broader bioethical debate on the subject matter.

## Methods

**Fig. 2:**
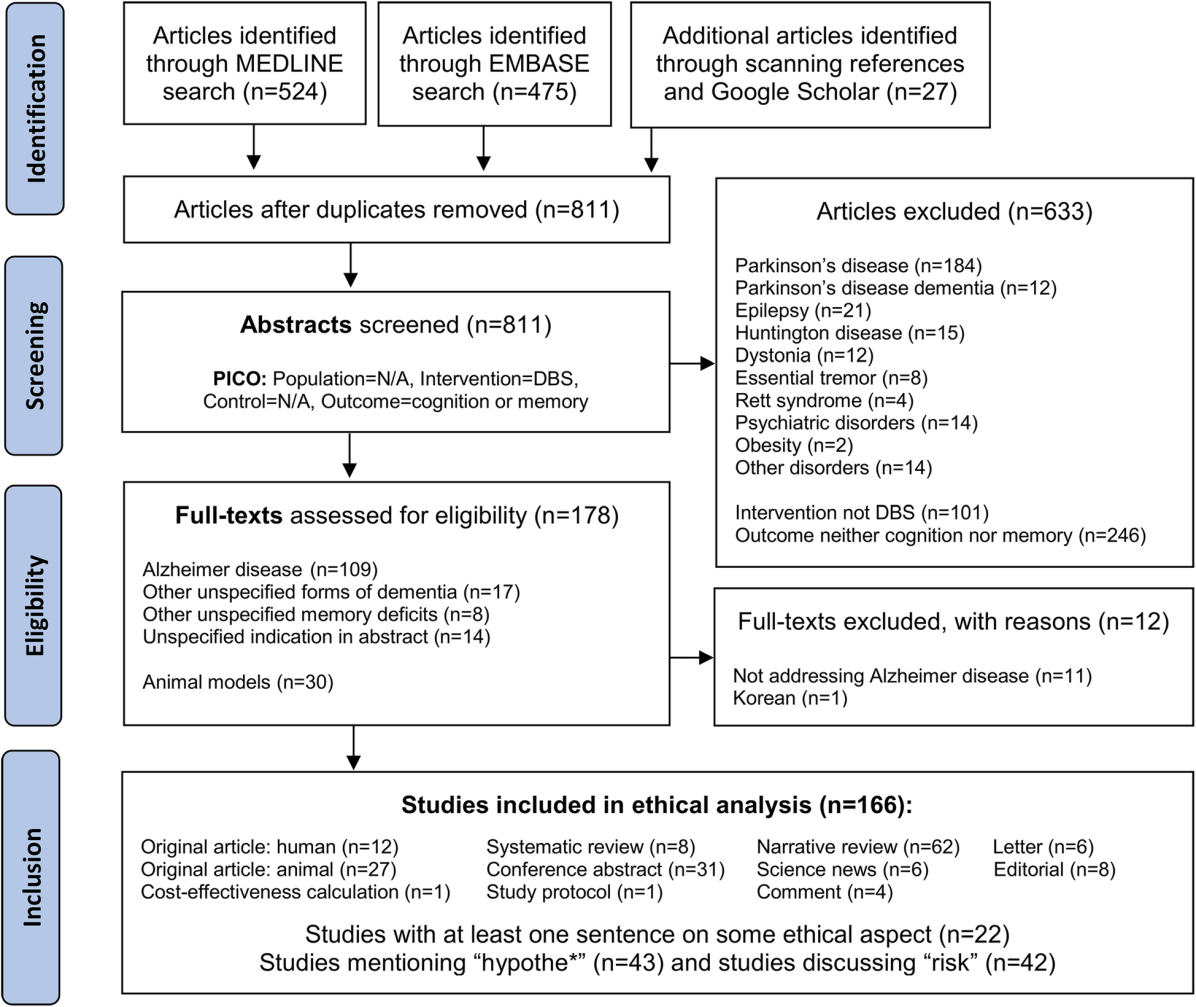
PRISMA flow chart [113] of the systematic literature search for DBS for cognitive function and memory

A systematic literature search was performed on several distinct databases. We searched MEDLINE using the PubMed interface and EMBASE via OvidSP as well as ClinicalTrials.gov, ChiCTR.org.cn, EnsaiosClinicos.gov.br and CENTRAL with the search terms “deep brain stimulation,” in combination with either “cognitive function” or “memory”. The results of this search were systematically kept up to date till May, the 4th 2017 using a MyNCBI e-mail alert for new publications matching the search terms. No search filters for language or publication type or research subjects (human versus animal) were applied and no limit on time period. We additionally screened the reference lists of included publications for further relevant articles and searched additional information using Google Scholar and an informal grey literature search (Fig. 2).

After having read the abstracts of 811 publications on memory or cognition and DBS, we examined 175 full texts for eligibility and included all but one of the retrieved publications that discussed DBS, Alzheimer disease (ICD-10 G30.*) and cognitive function or memory (*n* = 166). We categorized the articles for publication type and systematically assessed the topics “ethics”, “research rationale” and “risks”.

## Results

The search retrieved only a sparse amount of relevant empirical research on DBS for AD. There are 2.9 times more papers (*n* = 166) than patients (*n* = 58), and merely 7% of the publications (*n* = 12) report genuine primary data of patients receiving DBS for AD (Fig. 2). This indicates high interest of the scientific community but sparse primary research at the same time. Publications of lower methodological rigor like conference abstracts (*n* = 31) and narrative reviews (*n* = 62) were about 4 and 8 times more frequent then publications of high methodological rigor such as systematic reviews (*n* = 8).

To evaluate success or failure of DBS for AD research, long term evaluation of the disease progression is critical. Since its launch in 2008, the mean follow-up period for published clinical data is to date 13.5 months [14–16, 18, 19, 40, 41], with only seven patients for whom at least 24 months of partial follow-up data is available from Letters to the Editor [19, 40].

**Fig. 3:**
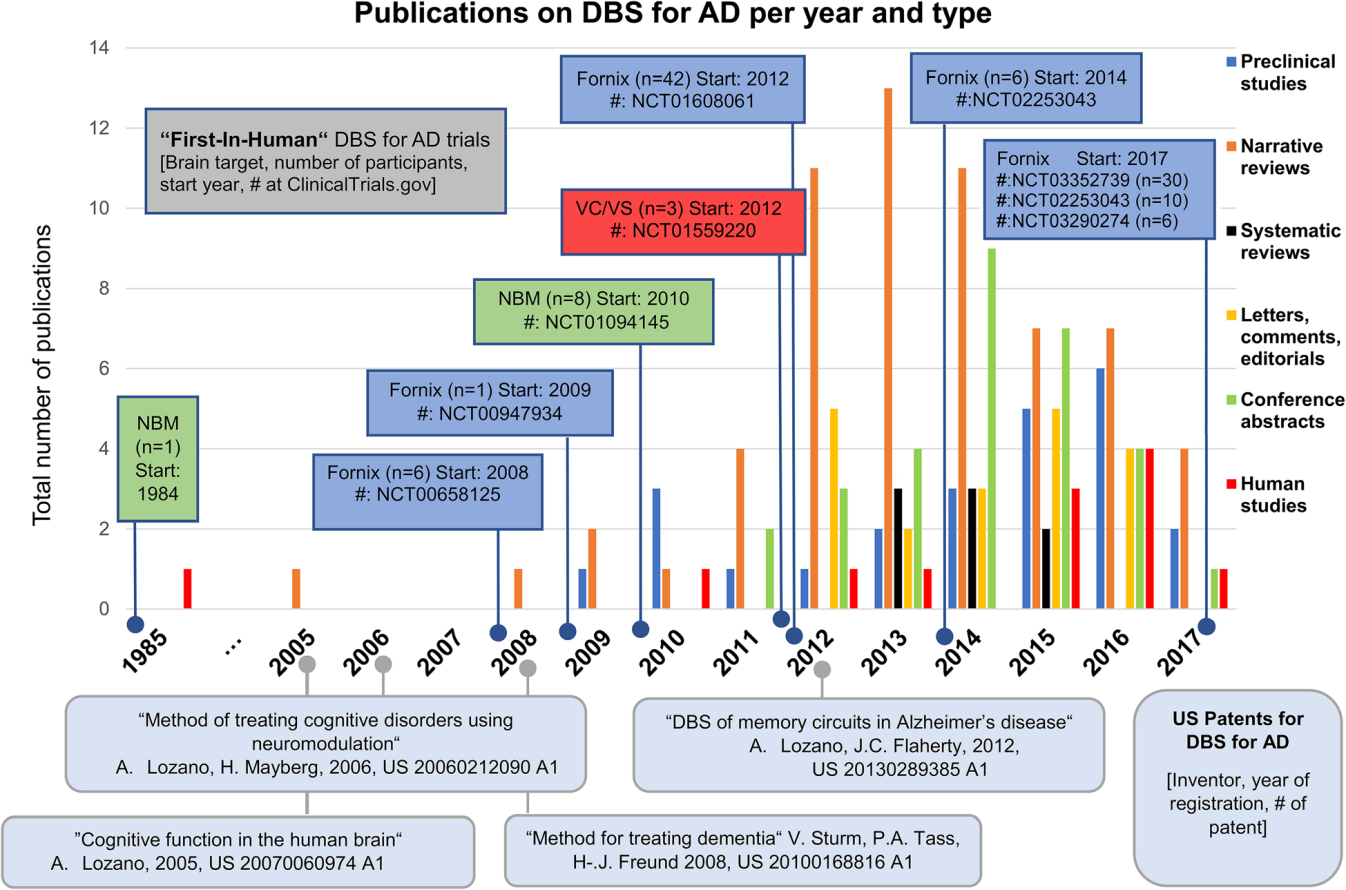
Results of the systematic literature search. Shown are the number of publications per year and publication type. In addition, patents on DBS for AD are depicted (oval boxes) as well as registered clinical trials on DBS for AD (rectangular boxes)

In total, we identified six registered clinical trials that had all started before a solid scientific foundation of DBS for AD was published and particularly before preclinical studies in disease-specific animal models were performed. It appears that human research triggered the surge of animal research and literature reviews rather than the other way around (Fig. 3).

Whereas the trials with sites in Canada, USA, Germany and France recruited mild to moderate AD patients, one Chinese trial (NCT03115814) reports to enroll six severely demented patients (MMSE < 10) [21]. However, for this trial, we could not find any published data on MEDLINE, EMBASE or the China Knowledge Resource Integrated Database. Furthermore, four US patents were granted and hold by DBS researchers in the field.

The systematic search retrieved 22 articles containing at least one sentence addressing some ethical aspect of DBS for AD, 41 articles discussing risks and 43 articles discussing research hypotheses. We clustered the results in three ethically relevant categories that are discussed in the following sections: 3.1 research rationale, 3.2 risk-assessment, and 3.3 interpretation of results.

We found grey literature evidence that additional patients have undergone fornix DBS for AD in Brazil and others are planned. Yet, no respective published material in scientific journals or clinical trial registries was found.^8^ Compared with the peer-reviewed published data of 58 patients [14–16, 18, 19, 41], we found a potential number of 75 yet to be published cases (see [20, 21] and endnote ^8^) that have never been discussed in the literature before or only published as conference abstracts (*n* = 3). These cases comprise the premature application of fornix DBS for AD to clinical practice in Brazil (*n* = 1), which was condemned as unethical by the Brazilian Academy of Neurology (see endnote ^4^). A small case series launched in the US in 2012 (n = 3), using a deviant target (VC/VS) compared to the rest of the field (fornix or NBM). As well as ongoing trials in China (*n* = 30, NCT03352739; *n* = 6, NCT03115814; *n* = 10, NCT02253043) and Spain (n = 6; NCT03290274) and a potentially unregistered trial (*n* = 17) announced by neurosurgeons via public media in Brazil (see endnote ^8^). Taken together, these cases represent a significant number of so far unpublished clinical data. As long as the clinical outcomes of some cases remain unpublished, the theoretical possibility of publication bias cannot be ruled out entirely. This poses the risk that the published data may not be representative for the field of DBS for AD research as a whole.

### Research rationale - open questions posing empirically unaddressed potential risks

From 2008 to 2012, the time when the so far published clinical trials on DBS for AD were launched,^9^ animal studies to better understand the mechanism of action of DBS for AD were scarce (*n* = 7). Not before 2014, did any animal study examine one of the different brain targets that are currently investigated in humans directly in an animal model of AD [42–50]. In addition, all of these studies were exclusively concerned with efficacy (NGF release: *n* = 2, hippocampal neurogenesis: n = 2, long-term potentiation: *n* = 1, short-term potentiation: n = 1, short-term memory: n = 1, or memory impairment: n = 2). None assessed primarily safety or toxicity of stimulation, although two mentioned major caveats (e.g. using stimulation “2–3 times higher” than the “safety threshold for human studies” [47] or that the effect was only found in adult rats but precisely not in aged rats, which would be closer to modeling AD [50]). The fundamental research question how the clinical neurophysiology of AD interacts with any potential beneficial DBS effect was examined in rats [51–56] only after the first experiments in humans had been performed [14, 57]. Still in 2017, there is a huge preclinical research gap examining animals specifically modeling some aspect of AD pathology in combination with relevant brain targets as well as examining the safety of stimulation parameters that are hypothetically clinically efficacious. This lack of evidence considerably exacerbates the translational challenges for human DBS for AD [57]. It includes the choice of best neuroanatomical target for placing the electrodes, the role of stimulation parameters and the assumed neurophysiological effects of stimulation. In consequence, very diverse candidates for the presumed mechanisms of action of DBS for AD were suggested.

### Uncertainty about the hypothetical mechanism of action

The proposed mechanism of actions of DBS for AD are as diverse as increasing cerebral glucose metabolism [14, 18, 22], “neural hijacking” by resetting theta activity [22], increasing hippocampal acetylcholine release [22], compensating “neuro-chemically for the cholinergic fibres which have already been lost” [58], enhancing neuronal activity [12], increasing nerve growth factor (NGF) release [22], alleviating functional and structural brain circuit aberrations [18] or “normalization” neural oscillations [8, 15]. Noteworthy, all discussed hypothetical effects of DBS in the fornix or NBM are presented as potential beneficial effects of stimulation (i.e. efficacy), whereas any potential toxicity (i.e. safety) or potential clinical irrelevance (i.e. futility) of DBS for AD received little attention. Despite this plethora of hypotheses on potential positive physiological effects only measures for two of them were reported as outcomes in clinical trials, i.e. cerebral glucose metabolism and functional brain imaging [14, 15, 18].

### Cerebral glucose metabolism in AD

All studies assessed the surrogate marker FDG-PET (secondary outcome) [14, 15, 18], which is a measure of neural energy demand and may indirectly reflect any changes of neuronal activity (hypothetical benefit). However, increased cerebral glucose metabolism is clearly of manifold etiology. No study thoroughly discussed alternative explanations or questioned their hypothesis given that “[g]lucose metabolism is elevated in inflammatory processes and infections, with consequently increased FDG uptake” [59], which may thus constitute a potential alternative explanation.

Furthermore, none of the preclinical studies we identified, demonstrated that DBS of fornix or NBM is clearly nontoxic if applied to AD brain tissue. And neither did the studies report measures assessing negative effects like amyloid deposition or tau pathology before 2017 [51]. According to our analysis, none of the clinical trials [14, 15, 18, 41] reported biomarkers for neurofibrillary tangles (FDDNP-PET) or amyloid deposition (PiB-PET) [60] to keep track of any stimulation-induced benefits as well as potential deterioration.^10^ Recently, a study using optogenetics showed that neuronal activity enhances tau propagation and tau pathology in vivo in a mice model of AD. The authors concluded that “there may be negative implications for stimulation therapies such as deep brain stimulation or transcranial magnetic stimulation that are currently in clinical trials for AD.” [61]

### Brain circuit pathology in AD

Another hypothetical beneficial effect of DBS for AD is the “normalization” of brain circuits [26], but no definite, specific, and pre-established criteria to distinguish pathological values from normal values have been cited in the clinical trials [14, 15, 18, 41] and to our knowledge there are no sensitive and reliable criteria yet to qualify brain circuit alterations unambiguously as “normalization”. Such criteria would seem essential to accurately evaluate the clinical significance of any changes brought about by DBS with regard to brain circuits. In consequence, the inclusion criteria of the first DBS for AD trials did not particularly select participants with regard to the several assumed hypothetical mechanisms of action.

For example, if it is assumed that DBS “normalizes” aberrations of cerebral glucose metabolism, then the inclusion of participants should rely on biomarkers that indicate “pathological” cerebral glucose hypo-metabolism by clearly stated entry criteria [62]. However, the question what amount of change in cerebral glucose metabolism can be considered to be clinically relevant to AD patients seems to be still open. None of the trials reported respective criteria for treatment success or failure as determined by predefined levels of clinical significance [14, 15, 18, 63, 64]. In consequence, research with human subjects [10, 14–16, 18, 63, 65] preceded translational research that might have pre-clarified open questions about the assumed mechanism of action and its potential efficacy and safety profile [66].

While there is value in therapeutic progress and innovation, there are perils in experimental adventurism. If trials are continued, although primary endpoints like cognitive function (ADAS-Cog) have not improved significantly (as “main effect”, not in subgroup post-hoc tests), one must expect clear and well-established criteria of clinical significance for measures of secondary endpoints like “brain circuit pathology”. This is necessary to avoid the charge of merely explorative or hypothesis-generating human experiments.

The mere variety of hypotheses already asks for further meticulous research efforts. Many of these questions may well be pursued in animal models of AD or with milder research strategies than DBS. This reasoning is shared by some researches working in the field of memory effects of DBS for epilepsy. Itzhak Fried commented that “it is still a mystery how massive 130 Hz stimulation of the fornix at currents of thousands of microamperes might affect these intricate networks and their deterioration. This is indeed a knowledge gap that has not been adequately addressed even in rodents” [67].

In 2012, 4 years after the first trials started in humans (Fig. 3), there was still only sparse preliminary knowledge from rodent studies [22, 57] examining efficacy of fornix or NBM DBS with a broad variety of stimulation parameters [17]. Even 1 year later, when a pivotal (“phase II”) trial already begun to enroll human participants [18], comprehensive reviews of the literature did not report any directly relevant results (same brain target, same stimulation parameters) from animal studies that specifically modeled the disease pathology of AD or at least examined aged mice [12]. The Kuhn group clearly acknowledged the paucity of preclinical evidence when concluding that preclinical studies so far “almost exclusively were observed in naïve animals not actually suffering from dementia, therefore lacking the structural damages as well as the pathophysiology typically underlying dementia.” [68] However, such studies would seem to be necessary to find out whether the preliminary effects found in healthy animals, e.g. NGF release or enhanced neuronal activity, can also be expected in animal models of dementia. Because in these animals, NGF release itself seems to be impaired rather than merely diminished [22] and increased neuronal activity may not be safe [61].

### Brain target selection for DBS in AD

It is entirely open which neuroanatomical structure is the best DBS target for relieving symptoms of AD [26]. Therefore, clinical trials on three distinct targets compete for participants: the fornix, the NBM and the VC/VS.

According to their own reasoning,^11^ the Lozano group proposed the fornix merely based “on a serendipitous clinical observation” in one single patient with an entirely different condition (morbid obesity) [69].

The Kuhn group suggested the NBM [12], a reasoning that was mainly built upon theoretical reflection on the anatomy and organization of the NBM [58]. However, this proposal was criticized as rash by peer neurosurgeons [66]. They rightly remarked that target selection should not be based on theoretical considerations alone, but rather empirically guided and supported by translational evidence in order “to keep us standing in the realm of real science” [66].

Finally, the third target VC/VS in the frontal lobe was proposed on similar theoretical considerations [70]. The authors propose that “DBS to specifically modulate the frontal networks has never been performed but a is logical treatment approach” [20].

### Stimulating nerve bundles in atrophied targets in contrast to inhibiting neuronal hyperactivity

Imaging biomarkers like volumetric correlations between e.g. fornix size and the extent of memory impairment in AD may be promising candidates to aid future AD diagnostics or prediction [71]. However, it is not enough to show that a memory-related brain structure degenerates in AD in order to straightforwardly warrant DBS [66]. The example of PD is illustrative here: The substantia nigra is degenerated in PD, but not a target of DBS; in contrast, subthalamic nucleus and globus pallidus internus are targets of DBS, although they are not degenerated in PD [66]. Roughly, the rationale in DBS for PD is to restore dopaminergic effects by modulation of local neuronal activity with a reversible lesion-like inhibitory stimulation effect on the dysfunctional “output” of the STN or GPi. This is the reversible lesion hypothesis [72]. In AD, one may similarly argue that DBS may increase the impaired release of acetylcholine. However, in contrast to PD, there is no brain target whose “inactivation” is known to result in cholinergic effects in addition to the available cholinergic medication. The theoretical consideration that low frequency DBS is both safe and has a beneficial stimulatory effect on cholinergic neurons must first be established empirically, before atrophied nuclei containing cholinergic neurons (e.g. NBM) should be directly targeted.^12^ In addition, the clinical symptoms of AD are much less correlated with the functional loss of the cholinergic system if compared with the tight association between PD motor symptoms and the dopaminergic system [73].

Despite these striking differences, a theoretical assumption that could promote unrealistic expectations of participants in DBS research for AD is the undifferentiated equation of DBS for AD and PD. For example, the Lozano group pointed out: “The hypothesis is that, just as DBS for the neurodegenerative disorder Parkinson’s disease alleviates symptoms by modulating pathological network activity, that DBS-f [read: DBS of the fornix] might similarly prove a clinically beneficial therapy for AD” [18]. To draw a close analogy of PD and AD largely ignores the neuropathological differences of the two diseases, as well as the differences in target, stimulation parameters, and hypothesized mechanisms of action.

Taken together, it is up to now unclear what differentiates “pathological” from “normal” alterations of large-scale networks present in AD. The clinical relevance of respective alterations seems therefore speculative. This is a decisive difference between DBS for PD and AD. This difference also finds its expression in the fact that no biomarker for AD is established as inclusion criteria in analogy to the levodopa-response check of DBS for PD. Furthermore, some proponents of DBS for AD see additional research benefits, because “[p]atients with DBS of the fornix represent a unique opportunity to test hypotheses concerning the role of large-scale networks in AD brain activity and response to pathological insults” [74]. This research strategy puts the cart before the horse. We suggest that AD patients who undergo risky nontherapeutic procedures to advance research should not be enrolled for secondary and tertiary hypothesis testing such as additional time in functional MRI scanners to elucidate large-scale networks in AD. We think it is unlikely that these patients will directly benefit from these additional research strains. Quite to the contrary, neuroimaging studies should elucidate the role and relevance of large-scale networks for AD, before vulnerable patients are enrolled on the assumption that DBS may restore “pathological” large-scale brain circuits as has been proposed [75]. We suggest that in DBS for AD protocols, patients should always be granted the opportunity to opt out from additional hypothesis testing for which no directly relevant medical benefit is likely for these very patients.^13^

### Risk assessment: The lack of disease-specific and target-specific preclinical evidence

Because DBS involves invasive neurosurgery as well as electrical stimulation of pathologically altered brain tissue, there are certain risks involved. First, there are risks of the procedure. In particular, the invasive neurosurgery and the stimulation of pathologically altered tissue in never before explored brain targets near the hypothalamus with unclear side-effect profile (Table 1). Second, there is the risk of trial futility, i.e. the risk that a study fails to reach primary and secondary endpoints, or the risk of futile participation of individual subjects, who just do not benefit. Such risks of futility are prima facie the higher, the less prior knowledge is available. In particular, lack of information about the clinically effective stimulation parameters (frequency, amplitude, pulse width), the best brain target, and the various hypothetical mechanisms of action may risk the enrollment of non-responders. Especially, while it is still unclear how the ample variance within all these factors interacts with the presentation of the disease-specific pathophysiology of individual participants. In order to mitigate the risk of futility, internal validity is an important ethical requirement for risky first-in-human clinical trials.

### Unspecific inclusion criteria as threat to internal validity

Internal validity is the adequacy of a research project to provide an unbiased estimate of the true association between intervention and observed outcomes. Can we trust that the patients’ different disease progressions observed after DBS are due to the intervention as opposed to inherent differences within the recruited sample? In light of this question, inclusion criteria play a decisive role in responsible study design. The more homogenous the sample of phase I and II trials, the fewer the number of participants required to reach the same level of statistical power. Therefore, the more homogeneous the sample of early AD trials, the fewer the number of AD patients exposed to the neurosurgical risks of DBS to achieve the same research benefit.

To reach such a homogeneous sample, empirical knowledge on the interplay of the pathology and the investigational intervention is indispensable to guide the development of specific inclusion criteria. The importance of prior knowledge from empirical research to guide participant selection is again well illustrated by the history of DBS for PD. In the case of PD, dopaminergic responsiveness has soon become the established key inclusion criterion [76]. This reflects the fact that, since the 1950s, the basal ganglia-thalamocortical mechanism underlying PD was targeted by lesioning the ventrolateral thalamic nucleus [77]. In the 1990s, lesioning was replaced by DBS in the pallidum, which appeared as a reversible substitute for pallidotomy. This knowledge of the underlying pathology of PD is exactly what is reflected in the inclusion criteria of levodopa responsiveness, which is the best predictor of DBS outcome in PD patients [78].

In contrast, the typical neuropathology of AD is rather loosely connected to aberrations of brain circuits [26] and has not yet informed the specification of precise inclusion criteria. This is an important difference from the clear picture in PD and the biologically plausible hypothetical mechanism of action of DBS for PD. The conjecture of aberrant brain circuits in AD is completely detached from the classical picture of neurofibrillary tangles and senile plaques [79]. It is a considerable deviation from the cell-physiologically more specific hypotheses like the amyloid cascade hypothesis of AD [80]. And it also differs from innovative routes like the neuroinflammation hypothesis of AD [81].

Clearly, restoring impaired memory and/or cognitive function of AD patients by applying electrical currents to millions of neurons within a target is scientifically ambitious and complex. Therefore, it seems implausible that mere exploration of the huge variety of factors – brain targets, stimulation parameters and patient characteristics – will be very efficient. Even the lucky identification of a marked effect in some patients would be overall ethically questionable, if the lack of such effect in other patients could have been prevented with a more principled, hypothesis-driven approach informed by prior preclinical knowledge. One way to mitigate this risk is to avoid heterogenous samples. Therefore, we have outlined elsewhere that special care with different subtypes like familial and early onset AD ought to be taken [82]. Yet, further confinement of the inclusion criteria is needed. At least, the selection of participants should be specific to the hypothetical mechanism of action in order to reduce the risk of futile research participation.

### Pre-clarification of open question with milder means

Taken together, there are numerous issues to be solved. Namely, developing appropriate inclusion criteria, improving internal validity and increasing information on potential harms and benefits to inform future research subjects. These pending questions are strong pro tanto reasons to perform further basic science as well as in vitro and in vivo preclinical research and to do so in the right chronological order.^14^ Alternative explanations and risks should systematically be excluded by means of factual experiment. As long as the efficacy of DBS for AD is not established with appropriate preclinical research and its safety has not been examined in specific AD models, DBS experiments in humans seem questionable and in tension with Art. 21 of the Declaration of Helsinki [35].

To counter a common reservation, sound basic science and preclinical research will not necessarily slow down the bench to bedside development of therapeutic innovation. The slow-down often occurs when a promising therapy candidate “dies” in expensive phase I and II trials. Investigational interventions that have built “stamina” through extensive preclinical testing will not only be more likely to survive the “valleys of death” but may also surmount them more swiftly [83]. The likelihood of success is increased, because open questions are pre-clarified and inclusion criteria are more restrictive. As a result, samples are more homogenous in ways relevant to the intervention as well as the mechanisms of action and the specific disease pathology. In turn, pre-clarification of the open questions promotes the internal validity of well-designed pilot and pivotal clinical trials. Lack of information on these factors threatens internal validity and eventually poses the risk of futile research participation.

### Interpretation of results – Avoiding unrealistic expectations

In this section, we meticulously examine the interpretation of results from the respective DBS for AD trials and prospectively evaluate whether the communication is appropriate to better inform potential research candidates in the future.

### The risk of unrealistic expectation by selective emphasis of individual outcomes

Banning selective publishing of only positive results has long been ethically demanded for DBS research [13]. The main reason is that “the overreporting of positive results and the underreporting of negative results lead to a distortion of available evidence that might harm patients” [13]. The same is true for publications that report both negative and positive results, but that selectively emphasize individual outcomes as positive in abstracts and discussion sections, while negative outcomes remain undiscussed numbers in tables.

In their six-patient pilot study, the Kuhn research group investigated DBS of the NBM for AD and found that “the quality of life […] dropped slightly” and the primary outcome, the Alzheimer’s Disease Assessment Scale – Cognitive Subscale (ADAS-cog), “worsened by an average of 3 points after 1 year of stimulation” [15]. The ADAS-cog is a standard outcome measure for cognitive assessment in AD treatment trials [84]. Total scores range from 0 to 70. The higher the score, the more severe the cognitive impairments. A four-point difference between treatment groups is considered clinically relevant in 6-month anti-dementia drug trials [85]. The Kuhn group came to the conclusion that “the present DBS approach might slightly improve or stabilize the AD-associated symptoms in some patients” [15]. However, because the individual outcomes in this study are very heterogenous, this conclusion is only appealing if considering the mean outcome and outcomes better than the mean to be representative for describing the overall study outcome. If not, the inverse argument is just as appealing. One could also have issued a warning that DBS may accelerate the disease progression by selectively emphasizing individual cognitive profiles below the mean change that deteriorated within 1 year of stimulation “by 19 and 8 points, respectively” [15]. Certainly, both interpretations seem scientifically questionable. To prevent therapeutic misconceptions and unrealistic expectations, selective discussion of positive results must be omitted when discussing pilot studies (e.g., discussion of mean changes and better than mean changes, while omitting that standard deviations are two-sided). This is particularly important for small pilot studies, because they are typically by design inadequate to assess efficacy in scientifically credible ways [86].

Below, we present three examples that demonstrate why it is ethically important to critically discuss alternative explanations and methodological limitations of heterogeneous outcomes. First, the Lozano and the Kuhn group presented their results as a “stabilization” or “slow-down” of disease progression insinuating a causal link. Under close scrutiny, however, the “slow-down” is the result of a comparison with a pharmacological meta-analysis that seems flawed to us rather than a true positive finding. Second, DBS researchers started to constrain patient enrollment on the basis of a post hoc subgroup analysis that we call into question. The patient selection of the subsequent pivotal trial (“phase II”) seems to have been negatively influenced by this post hoc inference. Third, numerous publications including narrative reviews on DBS for AD made general remarks on safety and efficacy. By design, the data provided by pilot studies (“phase I”) is insufficient to draw general conclusions in statistically valid ways. Instead, the perhaps less attractive questions of feasibility should have been discussed thoroughly.

### Is there a “slow-down” of disease progression attributable to DBS?

The Kuhn group reported 6 AD patients who received DBS of the NBM. As primary outcome, the ADAS-cog was evaluated. The authors proposed the following interpretation of their results:

“ADAS-cog scores worsened by an average of 3 points after 1 year of stimulation (95% CI = − 6.1 to 12.1 points, P = 0.5). This observation points to a rather slow disease progression, as only an increase of more than 3 points on this scale is considered clinically significant. Accordingly, this change was less pronounced than the increase in ADAS-cog scores observed in an investigation of 686 comparable patients treated with anticholinergic [sic: read cholinergic] medication; the ADAS-cog score of this cohort yielded an increase by 4.5 points per year. In comparison, the ADAS-cog scores of the six patients from the first DBS Phase-I study in the fornix increased by 4.2 points over 1 year.” [15]

This interpretation is suggestive, but the cited results do not “point to” anything. The difference between the mean ADAS-Cog score at baseline and 12 months is *not* significant (*p* = .5) and the confidence interval is large (− 6.1 to 12.1 points). This casts doubts whether the mean is statistically well-suited to adequately represent the heterogeneous results within this small sample (*n* = 6). Future patients can realistically only expect that their ADAS-cog score will, with 95% certainty, either decrease by up to − 6.1 points (“benefit”) or increase by up to 12.1 points (“harm”) after 1 year. The results are simply not conclusive with regard to efficacy. The authors’ interpretation that the result “points to a rather slow disease progression” seems speculative and may even raise false hopes if taken at face value.

In addition, the comparison with the pharmacological meta-analysis seems to be flawed in our opinion. The meta-analysis assessed the efficacy of cholinergic medication for AD and found a decline in ADAS-cog score of 3 points within the first 12 months of their study (see Table 2 in [87]). This is identical to the average decline in ADAS-cog score after 1 year of NBM stimulation, where participants also received cholinergic medication (see Table 1 in [15]). The reported 4.5-point decline per year seems to refer to the mean decline per year over 48 months in the meta-analyzed drug trials. But because the rate of cognitive decline accelerates over time [88], a mean decline over 4 years must not be compared to the mean decline within the first 12 months of a study.^15^

**Table 2:** Ethical issues in DBS for AD research with human subjects. Answering these questions is crucial for deciding whether some novel intervention is ready for clinical testing

Risk of compromised scientific validity	*Threats to internal validity:* Is the heterogeneity in small samples regarding age, gender, disease stage, brain atrophy, AD pathology and potential confounding variables to large? Are clear criteria to evaluate study endpoints predefined?	*Insufficient statistical power:* Is the sample large enough to validly conclude that there are no significant adverse effects when there are none observed (type II error)? Is the sample size sufficient to draw reliable inferences?	*Open questions:* Which is the best brain target? What are effective stimulation parameters? What is the hypothetical mechanism of action? What are potential disease-specific challenges?
Risk of insufficient feasibility	*Electrode insertion:* Is it technically feasible to reach the target with sufficient precision given the brain atrophy and vascular alterations or trajectory required?	*Recruitment:* Is the study feasible and cost-effective enough to recruit a sufficient sample for adequately powered phase I and II clinical trials?	*Stimulation:* Is the range of therapeutically effective stimulation parameters limited by potential side effects?
Risk of therapeutic misconception	*Regulation:* Are patients aware of the research context or is it masked as therapeutic “compassionate use” or “clinical innovation” instead of nontherapeutic research e.g. under FDA’s Investigational Device Exemption label?	*Interpretation of results:* Are published results appropriately presented, avoiding reference to any speculative benefits that may result in unrealistic expectations? Are alternative explanations properly discussed? Are conclusions drawn in scientific valid ways?	*Media hype:* Is early media coverage disseminating exaggerated benefit expectations to lay people and potential participants while the evidence on any therapeutic effect is still speculative?
Risk of undermined informed consent	*Lack of information on efficacy and safety:* Are there concordant, valid and disease-specific preclinical findings replicated in different animal models and species?	*Mild, moderate to severe cognitive deficits:* Are participants able to properly understand the potential risk of innovative nontherapeutic trials without established direct benefit?	*Progressive loss of autonomy:* Given the foreseeable incapacity to consent, which provisions are installed for the lifelong follow-up period (e.g. adaption of stimulation parameters, hardware exchange)?

Another comparison that also seems questionable to us was made in further publications, where the Kuhn group reported data of two additional patients [40]. In this publication, they present data of the Mini Mental State Examination (MMSE). The MMSE assesses cognition and scores range from 0 and 30 (the lower the score, the more severe the cognitive impairment). Only changes in MMSE of at least 2 to 4 points indicate reliable changes and small changes can only be interpreted with great uncertainty [89]. The authors report that the MMSE score of one patient declined about 2 points over 2 years, which they consider “clinically non-relevant, since comparable but pharmacologically treated patients with AD show a decline of 4 points per year” [40]. Closer examination reveals that the historical controls of the pharmacologically meta-analysis showed a MMSE decline of 4.7 points over 24 months (see Table 2 in [87]), which amounts to 2.35 points per year, not 4. Moreover, the meta-analysis also “found that, even after 4 years of follow-up, 11.4% (*n* = 78) of their sample had no clinically meaningful decline in MMSE score” [87]. This means that any finding of the absence of a significant mean decline after NBM DBS may also occur in patients only receiving cholinergic medication. Given the chances of selection bias in non-randomly, carefully selected and very small samples like DBS for AD trials, the authors should have critically discussed that the rate of natural disease progression may vary and that the positive outcomes of some of their participants are within the range of the natural disease progression. Moreover, if referring to the group mean at all, they should have highlighted that the mean decline after receiving DBS and cholinergic medication was not significantly different from just receiving the medication alone.

Similarly, the Lozano group, also reported a “slow-down” of AD progression following DBS. However, this was de facto reported as “a mean increase of 4.2 points in the ADAS-cog in the 6 dB patients over 12 months” [14]. This is just what one would expect at early disease stages in patients receiving only cholinergic medication ([90, 91] see table in [14]). Nonetheless, the Lozano group claimed that “particularly fornix and NBM DBS, have emerged as viable, potentially beneficial treatment modalities for AD” [24].

As long as the results of pilot studies (“phase I trials”) on DBS for AD are not discussed addressing all plausible alternative explanations, including well-known covariates like age, education and disease stage and clearly citing appropriately matched numbers for historical controls, the claim that disease progression is slower in DBS trials than pharmacological trials is speculative in our understanding. Such claims may even contribute to the risk that Institutional Review Boards and potential patients may not be adequately informed.

### Is DBS for AD more effective for patients at an early stage of AD?

Being the pioneers of DBS for AD, the Lozano group were also the first to examine whether DBS may be specifically effective in certain subgroups. This is noteworthy, because the sample size seems extremely limited to evaluate efficacy in the first place. They interpreted the results of their uncontrolled, open-label pilot study (*n* = 6) on fornix DBS for AD the following way:

“There is also the suggestion that less severely affected patients are perhaps more likely to benefit, as we speculate due to having more of the integrity of the circuitry preserved. The early evidence suggests a clear relation with less severely affected patients less likely to decline after DBS.” [14]

As the authors speculate, their results may suggest the following interpretation. The earlier in the AD stage DBS is performed (the lower the ADAS-cog score at inclusion), the better the patient’s outcome 1 year after DBS (i.e. the lower the increase in ADAS-cog score after 12 months). However, this finding may be spurious and may amount to nothing more than the well-established correlation between AD stage and disease progression. In addition, a thorough discussion includes also possible placebo effects (given the lack of case control design in this study), learning effects (given the lack of alternative test forms of the ADAS-cog scale) and ceiling effects in subscales (given the known insensitivity of some ADAS-cog subscales in early and mild AD stages) [74]. To neglect these factors may potentially provoke false expectations in younger AD patients.

The Kuhn group discovered the same possibly spurious finding ex post facto [19]. On the basis of a sample (*n* = 8), they concluded that the only slight decrease in MMSE score “indicate that NBM-DBS performed at an earlier stage of the disease and at younger age may have favorable impact on disease progression” [40]. This conclusion appears to be problematic because the authors did not perform a statistically convincing subgroup analysis [92].

Taken together, the “clear relation with less severely affected patients less likely to decline after DBS” [14] may well be largely explained by the fact that disease progression is slower at earlier AD stages and then non-linearly accelerates over time [91, 93]. The Lozano group used the alleged correlation to inform later patient enrollment: “Based on observations in the Phase I study that patients with the best-preserved cognition and brain circuits were better responders, we targeted patients with mild AD” [18]. Contrary to their hypothesis, a significant number of the respective 42 patients were negatively affected by DBS of the fornix, and particularly the younger the patients. Because the correlation was based on a post hoc analysis in a very small sample (*n* = 6) it seems not very credible to us given the recommendations from the literature [92]. For this reason, we think that both the Kuhn and the Lozano group should have better refrained from using the correlation for patient enrollment in order to protect patients against potential unnecessary risks. More trenchantly speaking, the DBS researcher Fried commented that “the greater decline in the younger AD patients who received fornix stimulation may serve as a warning that electrical stimulation can also have detrimental effect beyond the risks of surgery” [67].

### When are trials adequate to evaluate safety and efficacy in addition to feasibility?

The proper purpose of first-in-human studies with very small sample size (*n* < 10) is feasibility [86, 94]. How many patients can be enrolled who fulfill all inclusion criteria, who are willing to participate, who can tolerate the study procedure and who de facto complete the entire trial from start to end without serious adverse event? This information is crucial for later trials that need larger samples to attain their research goal (e.g. to evaluate safety and efficacy). Without such information, enrolling AD patients may risk futile research participation. If researches ran out of suitable participants somewhere in the middle of a trial, the trial fails by design to answer any definite research question.

In France, Fontaine and colleagues performed a comprehensive feasibility study on DBS of the fornix in patients with AD [41]. Only one of 110 patients screened completed the study. The authors self-critically concluded that “this approach did not seem to reach the expectations of mild AD patients” [41].

In contrast, our analysis of the literature revealed that the Lozano and Kuhn group discussed feasibility in their pilot studies [14, 15] with only very few remarks although there are several relevant aspects to be found throughout their manuscripts that may inform feasibility of any future trials. For instance, Kuhn and colleagues stated that the aim of their study was to explore “the technical feasibility of NBM-DBS in AD” [15]. The authors concede that in “most cases, it proved impossible to insert an electrode into the preselected target” [15], because of “degenerative or otherwise pathological vascular alterations” [15]. This problem likely remains in the future. Furthermore, two out of six patients had to be re-operated, and one patient needed the anxiolytic lorazepam during the stimulation phase of the trial [15]. Nevertheless, they “conclude that DBS of the NBM is both technically feasible and well tolerated” [15].

The typical purpose of pilot studies (clinical trials of “phase I”) is to evaluate feasibility, but the Kuhn group had declared the procedure to be feasible and safe already in advance [12]. They claimed that considering the “low complication rate in a cohort of approximately 100,000 Parkinson patients treated with DBS globally, DBS seems to be a feasible and safe treatment for patients with dementia” [12]. Feasibility and safety of a novel procedure must be established empirically. Under no circumstances can a priori theoretical arguments or analogies to PD replace empirical investigation given the blatant differences in clinical pathophysiology.

Nonetheless, the Kuhn group concluded that DBS of NBM for patients with AD “may be considered a safe procedure and apparently lacks significant stimulation-induced untoward effects”. This neglects the surgery-related complication rate of 33% (*n* = 2) and selectively emphasizes the rate of adverse effects (16,7%) during the stimulation phase (lorazepam *n* = 1). We claim that one cannot convincingly declare a procedure to be safe on the basis of six patients because studies with such small sample size lack the statistical power to detect adverse events that are not extremely frequent.^16^

## Discussion

Generally speaking, raising unjustified hopes is ethically problematic in experimental human research with cognitively impaired participants who suffer from a serious illness like AD for which no effective cure exists. Therapeutic misconception may already occur when participants of clinical trials on unproven treatments tacitly still hope for direct personal benefit from participation [95]. In contrast, phase I and II trials on unproven treatments yield realistically only research benefits like generating data for testing particular research hypotheses. It is crucial that potential subjects understand that their participation would be an act of altruism. Since “[a]ltruistic individuals volunteer for research because they trust their participation will contribute to improved health for others and that researches will minimize risks to participants” [9], it is ethically required to minimize any unrealistic expectations of potential subjects. Part and parcel of promoting realistic expectation is the modest discussion of results. This view is also shared by other commentators on ethical aspects for AD (e.g. [96]).

Furthermore, to minimize the risk to participants, experimental research on risky (e.g. FDA’s and EMA’s risk class III) nontherapeutic interventions requires strict hypothesis-driven rather than explorative research. In particular, the mechanism of action underlying the hypothetical and to date unproven treatment effects need to be well-established by antecedent evidence from preclinical research. Since AD is a progressive neurodegenerative disease, an increasing loss of autonomy is foreseeable in patients suffering from AD [97]. In consequence, the central role of informed consent for clinical research becomes even more critical. DBS requires continued hardware maintenance and adjustments of stimulation parameters and bears the life-long risk of hardware-related infection. The foreseeable cognitive decline and the life-long medical follow-up makes the initial informed consent to altruistically volunteer for experimental DBS research especially important. Unrealistic expectations and therapeutic misconceptions must be minimized and participants should receive evidence-based information about any risks and benefits. However, there are signs that this has not always been accomplished.

As an example, patient recruitment often involves public postings on official websites of university hospitals. The Kuhn group recruited patients with mild and moderate cognitive deficits for their pilot study [15] with a flyer, in which they stated: “It cannot even be excluded that DBS effects a slow-down of disease progression, e.g. through the release of neurotrophins. Clinically, we hope to effect an improvement in patients’ cognitive capacities”.^17^ Of course, one cannot exclude the theoretical possibility of beneficial effects from yet uninvestigated interventions. But neither theoretical possibility alone, nor hopes are sufficient to warrant risky clinical research on unproven treatments. It seems questionable to us whether potential participants reading this information have entirely realized that there is only scarce preclinical evidence for this hope reported in the literature (see endnote ^5^) and that NBM DBS for AD had only been done in a single case before, which dates back to 1985, and with no relevant effect on clinical symptoms [16].

Moreover, a German PET-study with ten AD subjects comprising also DBS for AD patients showed that “the patient’s comprehension of informed consent information was rather limited” [98]. In this study, the capability for informed consent was investigated before the patients underwent an additional PET scanning. The study showed that the risks of trial participation were poorly understood by most patients. Participants only provided 5 to 50% correct answers to questions on informed consent information [98]. In light of such findings, advertisement for clinical trial participation and media coverage should be very careful to avoid unwarranted hopes. All the more because patients with AD and their relatives may tend to seek remedy from novel experimental interventions out of desperation and despair.

Furthermore, the Lozano group report that at least in some cases DBS for AD was performed without personal consent of the patients, as was the very first case of NBM DBS for AD in 1985 [16]. The Lozano group declared: “Written informed consent was obtained from patients or surrogates” [14]. Due to the foreseeable loss of autonomy in AD, it is desirable to additionally obtain informed consent from caregivers as was in fact reported in later publications of the same research group [18]. If, however, participants cannot give informed consent themselves, e.g. due to cognitive deficits, their inclusion in exploratory trials on risky, invasive interventions with yet unproven benefits seems ethically problematic and in conflict with Art. 28 of the Declaration of Helsinki.^18^ Since ongoing trials in China report to enroll severely demented AD patients^19^ [21], the question how to obtain informed consent from research subjects with cognitive deficits in this context seems unresolved. In our opinion, the standards set by the Declaration of Helsinki [35] should not be undercut.

Taken together, clinical trials on risky interventions should fulfill highest standards of patient protection. As long as there is no clinical indication for DBS for AD and no empirically established direct benefit, written informed consent of the participant and careful provision to counter unrealistic expectations and therapeutic misconceptions is indispensable. Our analysis, shows that recent pilot and pivotal studies on DBS for AD have in some important respects fallen short of these standards. [14, 15, 18] Table 2 summarizes the ethical issues that our systematic examination of the literature revealed. We hope this article promotes further bioethical discussion about where exactly the line of demarcation distinguishing valid science from scientific adventures is to be drawn (Fig. 1).

## Conclusion

Given the disease burden of AD, there is a moral obligation for research to improve the healthcare of these patients. This obligation is intensified by the increasing societal costs that are associated with AD in the future. At some point, such research on novel and still unproven interventions must accept the trade-off between therapeutic progress and the risk of very early or first-in-humans clinical research. However, the question where to draw the line on the continuum between cautionary protectionism and experimental adventurism is an ethical rather than a scientific one and is up for bioethical debate. We examined the rationale and ethical justification of DBS as an investigational intervention for AD. This systematic analysis aimed to comprehensively assess all relevant publications on DBS for AD and to evaluate them on ethically relevant aspects. Given the ethical and methodological evaluation that we have put forward, the analysis of 166 full texts revealed shortcomings that raise important scientific and ethical aspects that warrant in our opinion further bioethical debate and meticulous inquiry.

The main conclusion of our evaluation is that, similar to pharmacological research [99], investigational DBS research must refrain from overstatements and speculative interpretations in order to adhere to the self-imposed commitment “to the highest scientific, clinical, and ethical standards” [100]. Regarding therapeutic misconception and unrealistic expectations [96], premature conclusions on safety and efficacy should not be disseminated on the basis of underpowered studies unsuitable to evaluate safety and efficacy validly. In particular, suggestions such as the claim that “less severely affected patients [may be] less likely to decline after DBS” [14] or that disease progression seems to be slower in DBS trials compared to big pharmacological [15] trials seem speculative to us given the results of our comprehensive, systematic analysis of the literature. In light of the small sample sizes the putative statistical associations may also reflect “statistical noise” or false positives findings. We suggest that such claims ought to be avoided to reduce the risk of unrealistic hopes of potential future research subjects. Sound preclinical research [101, 102] is required to answer open questions and to provide better evidence-based information.

Given the fact that DBS may well be evaluated in comparison to the natural progression of neurodegeneration or historical controls, follow-up data on the cognitive development of all DBS patients since 2008 should be continuously published to keep track of any individual beneficial stimulation effect as well as any harms. This should be made a prerequisite, if the already performed studies are cited as evidence to justify further investigation in future trials. It must always be stressed that absence of evidence is not the same as evidence for the absence of possible harms.

Finally, our ethical analysis revealed that at least in some cases informed consent was reported to be obtained from surrogates only. In our opinion this would raise serious concerns. In agreement with German law and the Declaration of Helsinki,^20^ we think that informed consent of each and every participant is indispensable for investigational first-in-human research on risky neurosurgical interventions such as DBS with presently unproven direct therapeutic benefit to participants.

As long as these ethical issues remain unresolved, DBS for AD cannot be considered ready for clinical testing with humans. Institutional Review Boards and equivalent research ethics institutions are encouraged to request careful and critical evaluation of all existing evidence and published materials, ideally in the form of high quality systematic reviews and, if suitable, meta-analyses.^21^ This is especially important for conditions like AD, where effective therapies are lacking and patients are more likely – out of sheer despair – to seek help from unproven investigational interventions [97].

To protect participants against premature hypes and hopes, researchers should refrain from speculative interpretations and strictly discuss results in light of plausible alternative explanations. This demand is important for the whole field of DBS, because shortcomings in one area might also harm the reputation of DBS research in other areas.

## Abbreviations

AD: Alzheimer disease
ADAS-Cog: Alzheimer’s Disease Assessment Scale-Cognitive Subscale
CENTRAL: Cochrane Central Register of Controlled Trials (http://www.cochranelibrary.com/)
ChiCTR: Chinese Clinical Trial Registry (http://www.chictr.org.cn/enIndex.aspx)
DBS: Deep brain stimulation
EMBASE: Excerpta Medica database (https://www.elsevier.com/solutions/embase-biomedical-research)
GPi: Globus pallidus internus
MEDLINE: Medical Literature Analysis and Retrieval System Online (https://www.ncbi.nlm.nih.gov/pubmed)
MMSE: Mini Mental State Examination
MyNCBI: Service of the National Center for Biotechnology Information to retain user information and database preferences (https://www.ncbi.nlm.nih.gov/account/)
NBM: Nucleus basalis of Meynert
NGF: Nerve growth factor
PD: Parkinson’s disease
STN: Nucleus subthalamicus

## Footnotes

1 DBS is a medicinal device of class III (FDA and EMA) and requires clinical investigation before marketing for any new indication, e.g. an Investigational Device Exemption (FDA). However, the specific regulation depends on national law of respective countries and is therefore different for the U.S.A., Europe, Australia, China, India and other countries. DBS may also be performed as “off-label” or “compassionate use” without strict scientific proof of efficacy and safety. However, the line between clinical research and experimental investigations is blurred if a series of cases labeled as “compassionate use” is formally registered as clinical trial, is published in peer-reviewed journals and statistically analyzed. If “compassionate use” becomes altogether indistinguishably from normal research, then the different labels are only of legal relevance, but not relevant for the context of research ethics. A controversial question is whether emerging new DBS indications may also be studied in the context of surgical innovation [2, 3]. Because we focus on the ethical aspects of research, not on the legal aspects of regulation, the analysis of different regulation frameworks is beyond the scope of this article.

2 Hamani et al. clearly state that the original purpose of their study was to treat obesity: **“**The effects of hypothalamic stimulation on memory shown here represent an unanticipated collateral effect in the context of a putative treatment for morbid obesity” [7]. In spite of this purpose of their case study, Hamani et al. do not evaluate the implications with regard to the “putative treatment for morbid obesity” in the main text ([7]), but instead discuss that “it may be possible to apply electrical stimulation to modulate memory function and, in so doing, gain a better understanding of the neural substrates of memory.” ([7]). Only in the Supporting Information in the appendix, the authors evaluate the apparent failure to treat obesity (“sm_file_ANA21295_2.doc”, see: http://onlinelibrary.wiley.com/doi/10.1002/ana.21295/full [Accessed: December 13, 2017]). In the Supporting Information, they report that after changing the parameter settings from high to low frequency stimulation, the patient subjectively reported “reduced food cravings and a decreased tendency to binge” ([7]). However, they continue by adding that “the patient purposely turned the stimulator off some evenings using the hand held controller because he had a desire to eat and he felt it might help him sleep” ([7]). In consequence, “nighttime binging activity returned as before surgery and he regained the weight he had lost.” [7]

3 This research group also sought media coverage already early in the clinical testing phase promising minimal risks and beneficial effects resulting from supposed cortical innervation through enhanced acetylcholine release. A video in German on DBS for AD is available: http://www.ndr.de/fernsehen/sendungen/visite/schwerpunkte/Wir-haben-bisher-gute-Erfahrungen-gemacht,visite6193.html [Accessed: January1st, 2018]. Moreover, a video on the PD dementia patient is available from: http://onlinelibrary.wiley.com/doi/10.1002/mds.23141/suppinfo [Accessed: January 1st, 2018].

4 The neurosurgeon Rodrigo Marmo da Costa e Souza performed fornix DBS in a 77-year-old man in the Hospital Napoleão Laureano in Paraiba. Marmo explained in the Brazilian TV (Globo) that fornix DBS for AD is a safe procedure and referred to the phase I study of the Lozano group as empirical evidence. Marmo reports that his patient wanted to sell his house to undergo surgery in Canada, but eventually received fornix DBS in Paraiba, Brazil. http://g1.globo.com/pb/paraiba/jpb-1edicao/videos/v/neurocirurgiao-fala-sobre-cirurgia-para-frear-a-evolucao-do-alzheimer/4707938/ [Accessed: January1st, 2018]. The Brazilian Academy of Neurology condemned the case in a notice of clarification as unethical human experimentation: “O procedimento em questão fere os preceitos da ética médica”. http://abneuro.org.br/comunicados/detalhes/713/nota-de-esclarecimento [Accessed: January 1^st^, 2018].

5 For instance, the review articles in 2012 and 2013 did not report any preclinical study that matches the animal model of the disease indication, stimulation parameters or brain targets [17, 22] which were applied in humans already in 2008. Even 2017, a systematic review did not report a single preclinical study that directly examined animal models of AD and matched the very stimulation parameters and brain targets [57] used in human trials [14, 18]. That the Lozano group also considers preclinical evidence important, may be indirectly inferred from the fact that they published one directly relevant animal study in 2017 [51], i.e. 7 years after the first human pilot study [14]. In our opinion, this is the wrong order: we suggest to first perform preclinical research, and then, only if the latter is convincing and successful, to proceed to clinical research with human research subjects (see section 3.1 for further information and our ethical evaluation).

6 One often supposed ethically relevant difference is that DBS acts locally in a specific brain target whereas neuropharmaceuticals act globally across the whole brain, wherever the drug’s respective target is distributed. Indeed, at higher levels of frequency and voltage, continuous DBS results in a lesion-like effect interfering with “pathological” brain circuits [72]. With lower frequency and voltage, DBS is thought to intervene at a cellular level effecting biochemical processes like increased neuronal activity or release of neurotransmitters or neurotrophins [72]. Therefore, acknowledging the powerful effects of different stimulation modalities, invasive DBS intervenes also systemically with various cellular processes of the brain and may thus pose risks resembling bioactive pharmacological agents.

7 Informed consent requires voluntariness, comprehension and sufficient information. The last condition can be threatened by lack of information as well as information overload. Basic science and preclinical research are required to avoid the former; high-quality systematic syntheses are needed to avoid the latter.

8 The neurosurgeon Eduardo Barreto of the Hospital da Unimed in Rio de Janeiro performed fornix DBS in one 56-year-old patient. https://oglobo.globo.com/rio/bairros/uma-esperanca-no-tratamento-do-mal-de-alzheimer-8836157 [Accessed: January1^st^, 2018]. Additionally, Rodrigo Marmo performed fornix DBS for AD in one patient and reports one case in Sao Paulo (see link to TV interview in endnote^4^ above). The neurosurgeon Manoel Jacobsen Teixeira (Faculty of Medince of the Univerisade de Sao Paulo) and Alexandre Amaral (Hospital Federal dos Servidores do Estado do Rio) were reported to plan a trial of fornix DBS for early onset AD with 17 participants all younger than 60 years old. http://www.fapeam.am.gov.br/marca-passo-cerebral-e-testado-contra-sintomas-de-alzheimer/ [Accessed: January 1^st^, 2018]. No trial was registered either on clinicalTrials.gov nor the Brazilian Clinical Trial Registry (http://www.ensaiosclinicos.gov.br/). [Accessed: May 5th, 2017].

9 In 2008: ClinicalTrials.gov Identifier: NCT00658125, see also: [14]. In 2009: ClinicalTrials.gov Identifier: NCT00947934, see also: [41]. In 2010: ClinicalTrials.gov Identifier: NCT01094145, see also: [15]. In 2012: ClinicalTrials.gov Identifier NCT01608061, see also: [18]. In 2012: ClinicalTrials.gov Identifier: NCT01559220, see also: [18]. In 2017: ClinicalTrials.gov Identifier: NCT03352739, no published information yet available. In 2017: ClinicalTrials.gov Identifier: NCT02253043, no published information yet available. In 2017: ClinicalTrials.gov Identifier: NCT03290274, no published information yet available.

10 Clearly, PET imaging, which requires the injection of a radioactive tracer substance, is itself a further burden on patients. However, the decision to measure PET as secondary outcome of potential efficacy (cerebral glucose metabolism) instead of potential safety (amyloid deposition or tau propagation) is at odds with the research goal of these “first-in-human” phase I and II studies to assess safety.

11 In a textbook chapter, the Lozano group explains about their own study in Toronto: “A 6-patient pilot clinical trial has been performed by the group in Toronto to test the safety and possible efficacy of DBS of the fornix in patients with mild to moderate AD. The initiation of the study was based on the serendipitous observation of stimulation induced memories and enhanced memory function in a single obese patient who had electrodes implanted in the hypothalamus. This patient experienced vivid déjà vu sensations that were time locked to the application of electrical stimulation.” [114]. The same explanation is found elsewhere [69]. In the original article the research rationale is elucidated by reference to the 6-patient pilot study mentioned above, theoretical considerations about the functional role of the brain target and comparisons to other indication that are only remotely related to AD: “The hypothesis is that, just as DBS for the neurodegenerative disorder Parkinson’s disease alleviates symptoms by modulating pathological network activity, that DBS might similarly prove a clinically beneficial therapy for AD.” [18]. In our opinion, all this information together does not seem to constitute appropriate justification for their research rationale and high-quality, replicated preclinical evidence should be required prior to clinical trials in humans. In particular studies that are directly tailored to the disease-indication, brain target and stimulation parameters. [14]

12 All DBS patients were on cholinergic medication and DBS must therefore cause add on effects to the effects of these medications. One hypothesis how this could work is that low frequency stimulation has an excitatory effect on cholinergic neurons of the NBM. However, consistent preclinical evidence from animal models of AD supporting this hypothesis were lacking when human trials were initiated in 2010. Only 6 years later, a study showed promising effects of NBM DBS on glutamatergic function and memory performance in an animal model of dementia, but no cholinergic effect. The authors conclude: “Therefore, the spatial memory improvement in the present study may have been affected by other factors rather than Ach [read: acetylcholine, i.e. cholinergic effects]” [56].

13 We refer to people diagnosed with AD as ‘vulnerable’ in the setting of complex first-in-human research. The reason is that having *(a) cognitive deficits* as well as *(b) a serious illness for which no effective treatment is available* may increase the risk to misjudge whether the potential harms are reasonable relative to the expectable benefits [115].

14 Although animal studies cannot settle all open questions [12], there are more than 10 distinct transgenic mice models of AD [116] as well as “natural” *Octodon degus,* ovine or canine models, which may all be used to answer open questions how DBS interacts on a neurophysiological level with Alzheimer-like pathology. Multimodal preclinical research replicated in different models and species can therefore provide empirical evidence for any physiological effect like e.g. NGF or acetylcholine release that are hypothesized to be beneficial. Therefore, preclinical research still remains a *conditio* sine qua non for novel, experimental research on risky procedures, notwithstanding the use of laboratory animals should be reduced to the necessary minimum.

15 An alternative explanation would be that the authors applied some sort of matching of their patients with a subset of patients in the pharmacological trial. However, for the respective comparison, there is no explicit mention of any “matching” procedure with respect to relevant variables such as age, time since diagnosis, education or baseline ADAS-Cog [15].

16 Neither the six-patient pilot study of the Kuhn group [15] nor of the Lozano group [14] reported a priori power calculations to detect safety issues or evaluate efficacy. However, the largest trial comprising 42 patients reported: “The study was exploratory in nature and not powered to detect a statistically significant difference between treatment arms.” [18] The separately published safety assessment of the latter pivotal trial did also not report any power calculation [103]. Thus, it seems safe to us to assume as a general statement that all studies including the single case studies by Turnbull and colleagues [16] and by Fontaine and colleagues [41] as well as the three-patient study by Scharre and colleagues [20] lacked sufficient power to detect statistical significant differences.

17 Although the authors mention neurotrophins in the context of a potential slow-down of disease progression in the recruitment flyer, the release of any neurotrophins was not measured in this study. Kuhn, J., Hardenacke, K., and Lenartz, D. (2010). Patient recruitment flyer. Department of Functional Neurosurgery and Stereotaxy, University of Cologne, Cologne, Germany. [15] Available from: http://neurologie-psychiatrie.uk-koeln.de/psychiatrie-und-psychotherapie/forschung/arbeitsgruppen/Neurobiologie-und-Neuromodulation-psychischer-Erkrankungen/leichtgradige-alzheimerdemenz/1flyer-forschungsgruppe-v2.pdf/at_download/file [Accessed: January1^st^, 2018]. In contrast, the Lozano group clearly warned on their recruitment website that “At this time, it is unknown whether patients will benefit from participating in this study.” http://www.advancestudy4ad.com/alzheimers.php [Accessed: January1^st^, 2018].

18 The reason is that research participation is not to be conflated with therapeutic treatment. Surrogate decision-making for research participation involving no direct personal benefit (e.g. like in feasibility studies with unproven therapeutic effects) is only warranted, if risks are minimally higher than standard care [35–37]. For the respective passage from the Declaration of Helsinki, see endnote ^20^ below. Depending on national law, personal consent may not be required in the case of “compassionate use” as therapeutic treatment attempt, however, that there is such a clinical indication for DBS for AD is questionable at present.

19 See also the information provided at trial registration: https://clinicaltrials.gov/ct2/show/NCT03115814 [Accessed: January1^st^, 2018.]

20 Art. 28 of the Declaration of Helsinki states: “For a potential research subject who is incapable of giving informed consent, the physician must seek informed consent from the legally authorised representative. These individuals must not be included in a research study that has no likelihood of benefit for them unless it is intended to promote the health of the group represented by the potential subject, the research cannot instead be performed with persons capable of providing informed consent, and the research entails **only minimal risk and minimal burden**.” ([35], emphasis added by MB. See also [37] and [36]).

21 We are currently preparing a systematic review to assess risk of bias in publication on DBS for AD in a standardized way using PRISMA, AMSTAR, GRADE and other checklists. For the protocol see [117]
